# Supplementary material for: Identification of Intercellular Crosstalk between Decidual Cells and Niche Cells in Mice
Source: Int J Mol Sci. 2021 Jul 19;22(14):7696. doi: 10.3390/ijms22147696 (PMC8306874; doi:10.3390/ijms22147696)
Supplement: Supplementary file 1 [file ijms-22-07696-s001.zip › ijms-1289998-supplementary/suppl/Figure-S1.pdf]

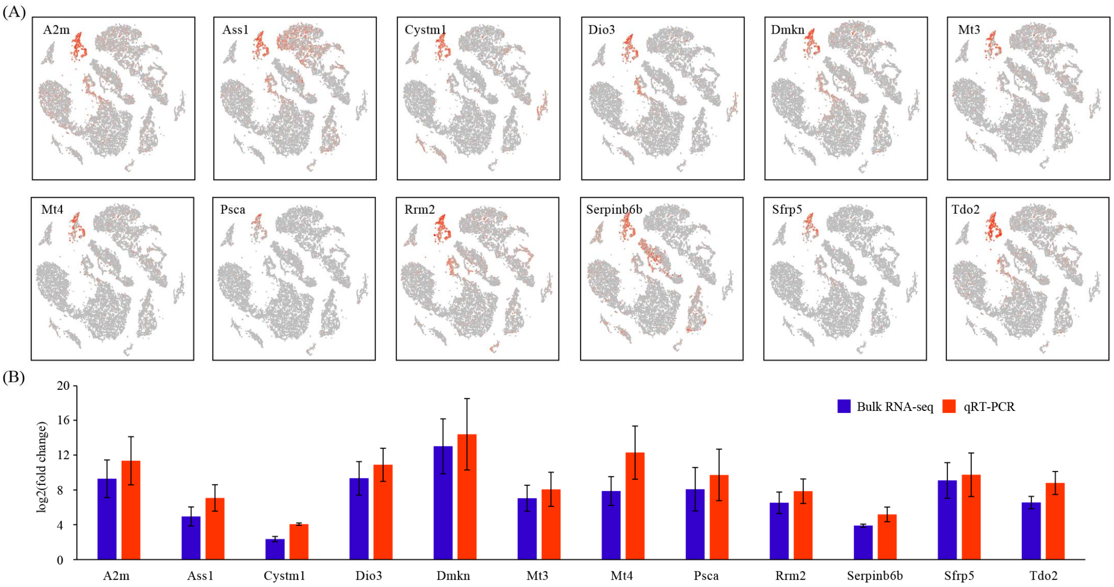

Figure S1. Validation of S3-enriched genes by quantitative RT-PCR. (A) TSNE plots showing the expression pattern of 12 selected genes. (B) Validation of selected genes by using qRT-PCR. Data obtained from qRT-PCR as well as bulk RNA-seq were displayed as mean ± SEM (standard error of mean). According to the t-test, $p < 0.05$ is true for all genes. n = 3.
