# Supplementary material for: Identification of Intercellular Crosstalk between Decidual Cells and Niche Cells in Mice
Source: Int J Mol Sci. 2021 Jul 19;22(14):7696. doi: 10.3390/ijms22147696 (PMC8306874; doi:10.3390/ijms22147696)
Supplement: Supplementary file 1 [file ijms-22-07696-s001.zip › ijms-1289998-supplementary/suppl/Figure-S2.pdf]

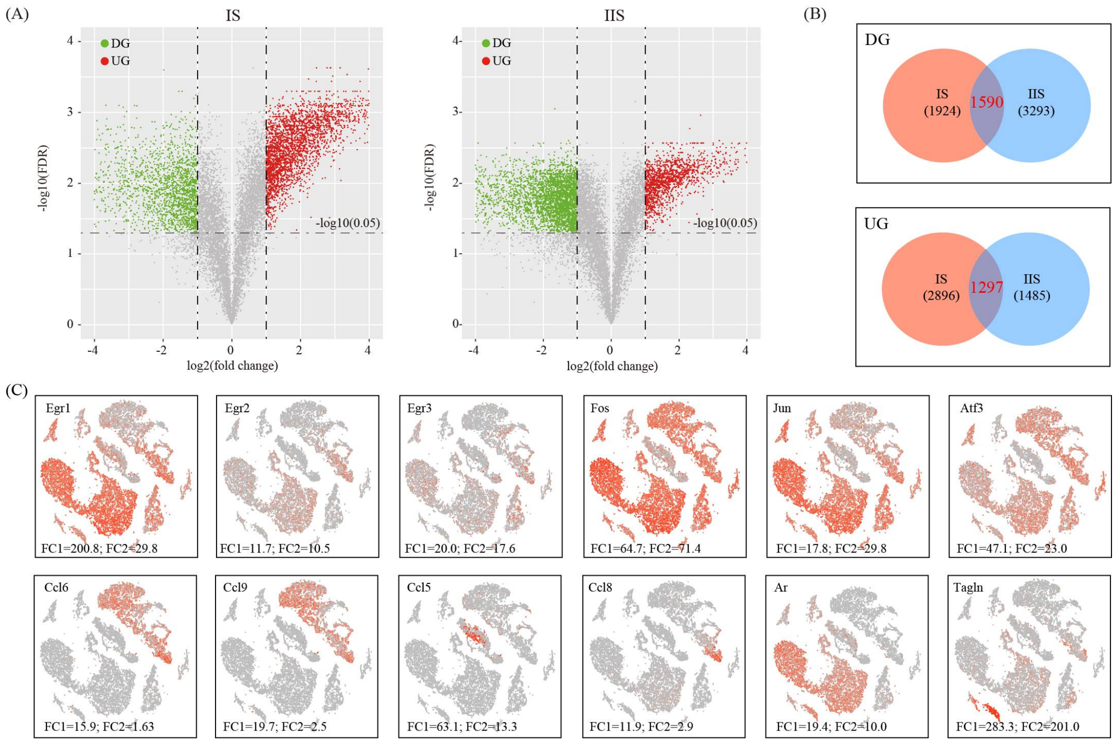

Figure S2. Comparison of single-cell RNA-seq data against parallel bulk RNA-seq data. (A) Volcano plot for the comparison between single-cell RNA-seq data and bulk RNA-seq data. IA and IIS were analyzed separately. Fold change >2 and FDR <0.05 were utilized to identify differentially expressed genes. DG, down-regulated genes; UP, up-regulated genes. (B) Venn plot showing the overlaps of differentially expressed genes. (C) TSNE plots showing the expression pattern of 12 selected up-regulated genes. FC1, fold change in IS; FC2, fold change in IIS.
